# The electrochemical reaction controlled optical response of cholestrol oxidase (COx) conjugated CdSe/ZnS quantum dots

**DOI:** 10.1038/s41598-020-77499-9

**Published:** 2020-11-24

**Authors:** Humaira Arshad, Madeeha Chaudhry, Shahid Mehmood, Ayesha Farooq, Minqiang Wang, A. S. Bhatti

**Affiliations:** 1 Centre for Micro & Nano Devices, Department of Physics, COMSATS University Islamabad, Park Road Campus, Islamabad, 45550 Pakistan; 2 Department of Bioscience, COMSATS University Islamabad, Park Road Campus, Islamabad, 45550 Pakistan; 3 Electronic Materials Research Laboratory, Key Laboratory of the Ministry of Education & International Center for Dielectric Research, Shaanxi Engineering Research Center of Advanced Energy Materials and Devices, Faculty of Electronic and Information Engineering, Xi’an Jiaotong University, Xi’an, 710049 China

**Keywords:** Optical techniques, Biochemistry, Biophysics, Biomarkers

## Abstract

This paper reports the enhanced performance of cholesterol oxidase (COx) conjugated CdSe/ZnS quantum dots (QDs) by using water-soluble mercaptoacitic acid (MAA) as linker. The functionalized MAA-CdSe/ZnS QDs conjugated in four different dilutions of cholesterol oxidase significantly affected QDs photoluminescence intensities, which affected the process of charge transfer from QDs to MAA. The conjugation of COx to MAA-QDs in increased dilutions resulted in the regain of PL intensities, which were attributed to the passivation of MAA HOMO/LUMO states. The electrochemical impedance spectroscopy and cyclic voltammetry of the conjugated QDs were performed to get study the charge transfer mechanism. The 1:1000 diluted COx conjugated MAA-CdSe/ZnS QDs was found to have the lowest charge transfer resistance of 228 Ω, the highest diffusion (~ 1.39 × 10^–13^ cm^2^/s) and charge transfer rates (~ 4.5 × 10^–6^ s^−1^) between the electrode and the redox species. The current study demonstrated the sensitivity of electrochemical and optical based detection on the alkaline.

## Introduction

Quantum dots (QDs) have been used extensively for numerous applications ranging from photonics to biology, e.g., light emitting diodes^1^ and bio-labels^2^, etc. The quantum dots are highly fluorescent materials as compared to the organic dyes due to high photostability and better lifetime. Furthermore, the dimensions of QDs can be made comparable to the biological molecules, e.g., proteins. The core–shell QDs, e.g., CdSe/ZnS QDs have been used as biomarkers in the study of bio-chemical reactions where QDs act as luminescent dye attached to antibodies. In this way, conjugated quantum dots are used for the detection of various diseases, most importantly cancer at an early stage. The conjugation happens when an electron is donated by the QDs to enzymes^3^. The photoluminescence of conjugated QDs with various biomolecules has led to the study of the structure of antibodies. The CdSe/ZnS QDs conjugated with various different enzymes like glucose oxidase are used for glucose sensing. The core–shell QDs have also been used for the detection of urea in the presence of urease; a catalyst. The degradation of urease directly affected the photoluminescence of conjugated QDs, which were used in the detection of urea levels^4^. The CdSe/ZnS QDs have also been used as a bioactive fluorescent probe in various diagnostic applications due to conjugation of protein molecules^5^. Semiconductor QDs like CdSe/ZnS are ideal systems for sustained long duration optical characteristics as it helps to go through the biochemical reactions occurring in real time and bast suited for in-vitro studies. The quantum efficiency is much higher than chemical dyes and tunability of colors gives a wide range of wavelengths in the visible region of the spectrum.

It is well known that the surface of core QDs is greatly influenced by the photophysical and photochemical processes. The disrupted surface creates surface defects to trap charges, which provides the non-radiative recombination routes and degrades the luminescence^6^. In order to preserve the luminescence, an inorganic layer of the shell is usually grown as the passivation layer, e.g., ZnS layer to cover CdSe QDs^7^. CdSe nanocrystals have demonstrated multiple emission bands mainly due to interband recombination, and participation of band gap defect states (surface defects) and deep traps created due to Se^2–^ rich facets. The deep trap emissions are due to holes trapped on the surface primarily residing in the selenium sp^3^ orbitals. The shell growth on QDs has shown to increase the quantum yield by almost 90%^8^. A variety of core–shell QDs (namely, CdTe/CdS, CdSe/ZnS/SiO_2_) have also been synthesized for biological applications. However, CdSe/ZnS QDs considered the best among other core–shell QDs for biological applications^9^.

Quantum dots synthesized via organic route require ligand exchange for biocompatibility with the biomolecules. Among different ligands used for biofunctionalization of QDs mercaptoacetic acid (MAA) is the most preferred one because of its ease of attachment to ZnS and its solubility in water^10^. MAA has a carboxyl group at one end which is helpful in conjugation with protein molecules (e.g., antibody). The thiol group at the other end is helpful in attachment to any sulphur containing molecule like ZnS^11^. So they can replace the trioctylphosphine oxide (TOPO) and bind to the surface of the QDs^12^. The attachment of thiol group results in the quenching of luminescence due to the formation of thiolates in the water accountable for the creation of hole trap states^9^. The quenching of the PL intensity is attributed to the charge transfer from the thiol group or carboxyl group to the surface of the QDs. The process of charge transfer makes the surface of QDs negatively charged. Thus, the deprotonation of thiol group is ensured by maintaining high pH value of the solution for the enhanced surface coupling.

Cholesterol oxidase (COx) is a bifunctional enzyme used to oxidize cholesterol in an isomerization reaction. It oxidizes and hydrolyses the cholesterol ester and produces the color producing hydrogen peroxide (H_2_O_2_) as a byproduct. The concentration of H_2_O_2_ in the reaction is proportional to the concentration of cholesterol in the serum^13^. The conventional cholesterol detection methods commonly used are liposcan, the lipoprotein profile^14^ and vertical auto profile (VAP) methods. Direct measurements of low-density lipoproteins can be made by ultracentrifugation. Similarly, VAP can be used for additional measurements of lipoprotein subclasses. However, such techniques are expensive and time consuming and as such commonly not accessible in cholesterol test settings.

A number of cholesterol diagnostic techniques have been established with increased sensitivity and selectivity^15^. The available cholesterol sensors are based on both electrochemical and/or optical detection^12,16^. The electrochemical sensor involves complex assembly with three electrodes. Alternatively, optical technique, commonly used for biological sensing, depends on the interaction between light and analytes. Among the optical detection methodologies, enzyme-based sensing is advantageous, which involves oxidation of target biomolecule and provides the reusability of the sensor. Because of inadequate filling capability and bioactivity of enzymes, nanostructured materials have been used^17^. Semiconductor QDs have also been used in biomedical applications like the cholesterol level detection via lab-on-chip devices.

In the present work, core–shell (CdSe/ZnS) QDs were synthesized via the organometallic method by using TOPO as organic ligand and later made water soluble by replacing TOPO with MAA. The CdSe/ZnS QDs were conjugated with different dilutions of cholesterol oxidase enzyme via EDC and NHS and the effect of conjugation in various dilutions on the photoluminescence (PL) intensities of QDs was studied. The electrochemical impedance spectroscopy (EIS) and cyclic voltammetry (CV) was employed to get an insight of the chemical reaction that affected the PL intensity in various dilutions. The EIS and CV results were analyzed to determine the route and efficiency of charge transfer.

## Materials and methods

### Chemicals

Cholesterol oxidase (COx, EC 1.1.3.6 from *Streptomyces* sp.), cadmium oxide (CdO, 99%), toluene (99%) trioctylphosphine oxide (TOPO, 98%), octadecylphosphonic acid (ODPA, 97%), trioctylphosiphine (TOP, 97%), octadecane (ODE, 98%), thioglycolic acid (TGA/MAA, 98%), 1-ethyl-3-(3-dimethylaminopropyl) carbodiimide (EDC, 98%), *N*-hydrooxysulfo-succinimide (NHS, 98%), cyclohexylisothiocyanate (CYS, 98%), zinc stearate (25 g) of analytical grade were purchased from Sigma-Aldrich (USA). All chemicals were used without any alterations.

### Synthesis of CdSe QDs

CdSe QDs were synthesized as in the following. CdO (0.06 g), TOPO (2.50 g), ODPA (0.28 g) and ODE (2 ml) were first mixed at 150 °C in vacuum for 1 h in a three-neck flask. The temperature was then raised to 340  °C for the dissolution of CdO. Once the solution turned colorless, the Se-TOP mixture of Se (0.058 g) in TOP (1 ml) was injected and the reaction was carried out for another 20 min. The reaction was quenched by the addition of toluene (1 ml) at 60  °C to prevent its solidification. The CdSe QDs were purified in isopropanol (IPA) and methanol via centrifugation (@3000 rpm for 30 min each).

### Synthesis of CdSe/ZnS core–shell QDs

The growth of ZnS shell was done using the recipe given in^18^ with a slight modification. TOPO (2.50 g) was heated at 150 °C in vacuum in a three-neck flask. Then TOP (0.5 ml) was injected at 60 °C and temperature was raised to 100  °C before adding 36 mg of CdSe QDs. The prepared solutions of zinc stearate (0.127 g) (prepared in TOP (0.75 ml) and ODE (0.5 ml)), CYS (120 $\upmu$l) (mixed in ODE (1 ml)) were added at 240 °C. The reaction was performed at 280  °C for 20 min. Finally, toluene (1 ml) was added at 60  °C to prevent its solidification, which was followed by purification of CdSe/ZnS QDs with isopropanol (IPA) and methanol.

### Preparation of cholesterol oxidase (COx) dilutions

A stock solution of COx was prepared in phosphate-buffered saline (PBS, pH 7.4) and then serial dilutions prepared from this stock were 1:10, 1:100 and 1:1000.

### Conjugation of CdSe/ZnS with COx antibody

**Figure 1:**
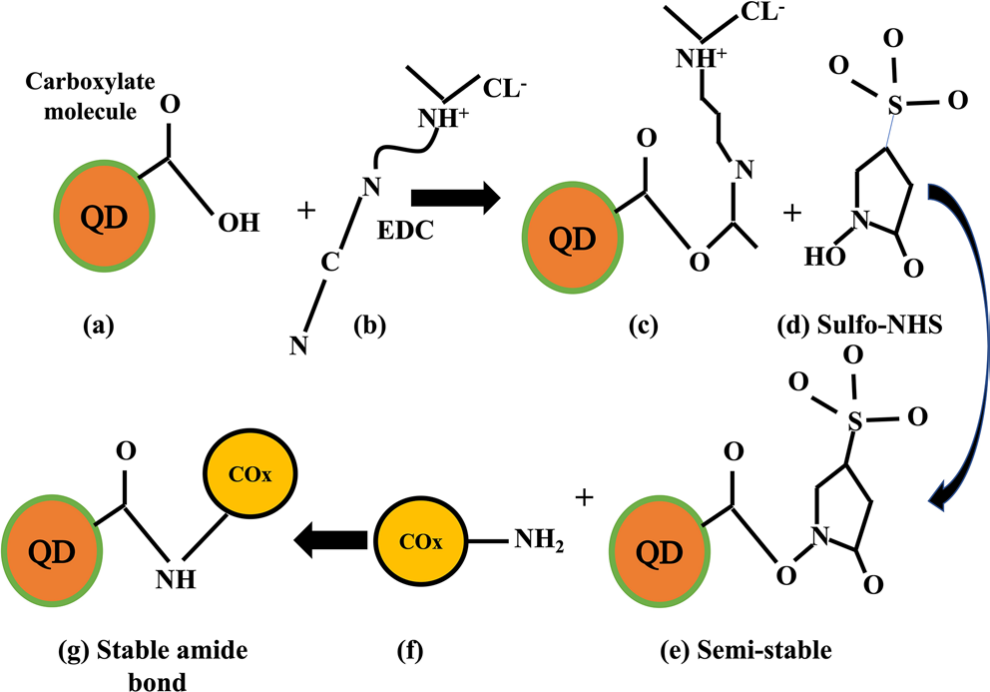
The schematic representation of MAA capping of CdSe/ZnS QDs and conjugation of Cox with CdSe/ZnS QDs via EDC/NHS in various steps a-g as discussed in the text. The steps involved are (a) formation of carboxylate bond via thioglycolic acid; (b) addition of EDC for activation; (c) unstable reactive ester formed; (d) stabilizer added; (e) semi-stable amine reactive NHS ester; (f) cholestrol oxidase addition; and (g) COx conjugated QD.

The scheme of synthesis of COx conjugated CdSe/ZnS QDs is shown in Fig. 1 (steps a–e). The CdSe/ZnS QDs were made hydrophilic or water-soluble by dispersing QDs and MAA (step a) in equal quantity in toluene and left for half an hour for the reaction. The upper layer of water soluble MAA coated CdSe/ZnS QDs (pH 3.6) was separated by centrifugation (@ 3000 rpm for 10 min) and then dispersed in distilled water for the attachment of COx antibody. The activation of –COOH end of the CdSe/ZnS-MAA QDs was done by using EDC (5 mM) (step b) thus, produced unstable reactive ester (step c). Further, the addition of NHS (1 mM) resulted in the formation of semi stable amine reaction ester (step e). For carboxyl activation and formation of amide bond the reaction was left for 10 min. The freshly prepared COx solution (stock solution) was added to activated MAA coated CdSe/ZnS QDs (step f) and left for conjugation for 12 h, which produced COx conjugated MAA-CdSe/ZnS QDs at pH 4.5. The pH of MAA coated dots as mentioned earlier was 3.6; while for EDC/NHS the solution pH was adjusted at 4.5 by using 1 M NaOH. The conjugation occurred due to the formation of stable amide bond between NH^+^ present in COx and COO^−^ in MAA–CdSe/ZnS QDs (step g). The same procedure was repeated for other dilutions of COx (1:10, 1:100 and 1:1000). The recorded pH for 1:10, 1:100 and 1:1000 diluted COx conjugated CdSe/ZnS QDs were 4.53, 5.56 and 6.07, respectively.

### Preparation of the bioelectrode

The bio-conjugated electrode was prepared by modifying the surface of glassy carbon (GC) electrode for electrochemical studies. The GC electrode was cleaned by sonication in acetone followed by alumina slurry coating of electrode, which was cleaned in sulfuric. Finally, the cleaned GC electrode was modified with CdSe/ZnS QDs (10 $\upmu$l) dispersed in toluene (pH 10.2). The CdSe/ZnS QDs modified electrode was then rinsed with DI water and was dipped in MAA (10 $\upmu$l) for 30 min for MAA capping of CdSe/ZnS QDs (pH 11.3). The MAA–CdSe/ZnS modified GCE was then treated with EDC and NHS solution to functionalize COOH group. The EDC/NHS modified GC electrode was then dipped in COx stock solution for conjugation of COx antibody with CdSe/ZnS QDs attached to the electrode (pH 10.2). The process was repeated for all dilutions of COx to conjugate COx with MAA–CdSe/ZnS QDs. The recorded average pH values of GCE after modification with different COx dilutions were 10.3, 10.5 and 10.8 for 1:10, 1:100 and 1:1000 dilutions, respectively.

## Characterization

The photoluminescence (PL) spectra of the conjugated QDs was collected using LabRAM spectrometer by Dong-Woo Optron. He-Cd laser was used for excitation emitting at a wavelength of 325 nm with a power of 50 mW. The scattered signal was detected by triple grating monochromator in the range of 350–700 nm. The PL spectra were corrected with the detector response. The electrochemical measurements were performed by electrochemical analyzer (Autolab, Metrohm 85695) equipped with three electrodes; glassy carbon electrode (GCE) acting as working electrode, platinum wire as counter electrode, Ag/AgCl as reference electrode. The data analysis was performed using NOVA (1.10.1.9) software for CV and frequency response analysis (FRA). The measurements were performed using 0.05 M K_4_[Fe(CN)_6_] and 3 M aqueous KCl electrolytic solution. The CV measurements were performed in the potential range from − 0.5 to 0.8 V at various scan rates from 0.03 to 0.13 Vs^−1^ with a step size of 0.01 Vs^−1^. The EIS measurements were also performed in the frequency range from 100 kHz to 0.1 Hz.

## Results and discussion

### Photoluminescence spectroscopy

**Figure 2:**
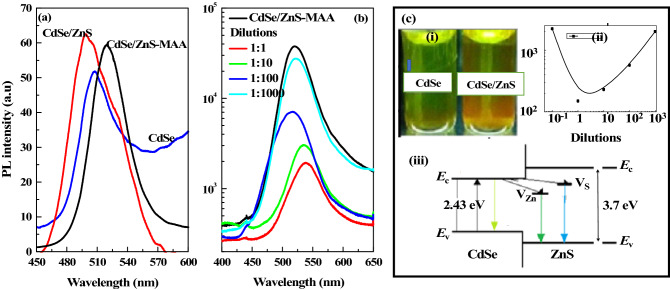
(**a**) Photoluminescence spectra of CdSe (blue color), CdSe/ZnS (red color) and CdSe/ZnS-MAA (black color). The inset shows a luminescence image of Left: CdSe QDs and Right: band diagram of CdSe/ZnS QDs. (**b**) The PL spectra of MAA-QDs (black color) after conjugation with different dilutions of COx: 1:1 (red), 1:10 (blue), 1:100 (green), and 1:1000 dilution (pink) COx conjugated QDs. (**c**) (i) The fluorescence images of CdSe (left) and CdSe/ZnS (right); (ii) plot of variation in the integrated intensities as a function of dilution; and (iii) the energy band diagram of CdSe/ZnS heterostructure with possible optical transitions from V_Zn_ and V_S_.

The PL spectra taken from the bare CdSe (black), CdSe/ZnS (red) and MAA conjugated CdSe/ZnS (blue) are plotted in Fig. 2a. The PL peak observed at 510 nm (2.43 eV) from the bare CdSe was also used to estimate the size of the QD, which was around 33 ± 2 Å. The asymmetry of the peak was attributed to the presence of surface defects^18^. The ZnS capped CdSe QDs showed enhanced PL intensity and a blue shift of 11 nm in the PL peak observed at 498 nm with increased FWHM. The blue shift in the peak position was due to passivation of band edge defect states of CdSe by ZnS capping layer. The increase in the PL intensity was the result of the improved quantum confinement.

MAA conjugated CdSe/ZnS QDs showed a minute drop in intensity and a red shift of 10 nm with the PL peak (black spectrum) at 519 nm as shown in Fig. 2a. The drop in the intensity was attributed to the loss of photo-excited carriers in QDs possibly to the HOMO/LUMO levels of MAA^11^. The drop in the intensity was related to the surface coverage of QDs with MAA^19^. The dissociation of small fraction of MAA from QD’s surface in low pH value led to reduce the surface charge^20^, which resulted in the aggregation of QDs and quenched the PL intensity^21^ and red shifted the PL peak position^22^. Figure 2c—(i) shows the fluorescence images of the bare CdSe (greenish) and CdSe/ZnS (yellow-greenish) QDs, It is very important to revisit the energy band diagram of the core–shell (CdSe/ZnS) QD as shown in Fig. 2c—(iii). ZnS is a wide band gap semiconductor and has optically active defect states in the band gap due to available S and Zn vacancies (V_S_ and V_Zn_) emitting at 440 nm and 520 nm, respectively. These states when resonant with the highest occupied molecular orbital (HOMO) and lowest unoccupied molecular orbital (LUMO) levels of MAA and behaved as mediator for transfer of charge from the QDs to bioactive material^10^.

The MAA-CdSe/ZnS QDs were then successfully conjugated with different dilutions of COx antibody. The PL spectra recorded for each dilution of COx conjugated MAA-QDs is plotted in Fig. 2b, which showed strong dependence of PL intensity on the dilution, which was ascribed to pH of the dilution. The recorded pH value for each dilution, i.e., 1:1, 1:10, 1:100 and 1:1000 was 4.50, 4.53, 5.56 and 6.07, respectively.

There was a sudden drop in the PL intensity of MAA-QDs after incubation in 1:1 COx dilution. In case of 1:1 dilution with pH in the range of 4.50–4.53, there was presence of protons in the media. The protonated groups (COOH, NH_2_^+^) in COx structure attracted the photo-excited electron in QDs and thus quenched the PL intensity. However, the media became alkaline on increasing the dilutions, and as result there was an enhancement in the PL intensity of MAA-QDs. This behavior was attributed to the deprotonated state of amino group, which did not attract the photo-excited electrons and thus enhancement in the PL intensity was observed (Fig. 2b: red and blue color). The presence of electron donating groups of Ab also passivated the electron trapping sites, i.e., LUMO of MAA^10,11^. As in case of 1:1000 dilution (Fig. 2b: pink color) COx conjugated MAA-CdSe/ZnS QDs, where the media was alkaline, which passivated the electron trapping sites of MAA and helped in the regain of PL intensity. This was ascribed to the charge transfer mechanism in COx conjugated MAA-CdSe/ZnS QDs^22^. In case of 1:100 dilution, the enhanced PL intensity as compared to other dilutions was ascribed to the presence of equal number of protonated and deprotonated groups of Ab in the media. This could also be due to increase in the passivation of surface defects, which resulted in decrease in surface charges and polarizability of the molecule^23^. Figure 2c—(ii) shows the plot of integrated intensities from Ab-conjugated MAA-QDs as a function of dilution. The increase in the PL intensities with the increase in dilution was ascribed to the charge transfer from and to QDs to MAA and to Ab. This was studied further by electrochemical impedance spectroscopy.

### Electrochemical studies

**Figure 3:**
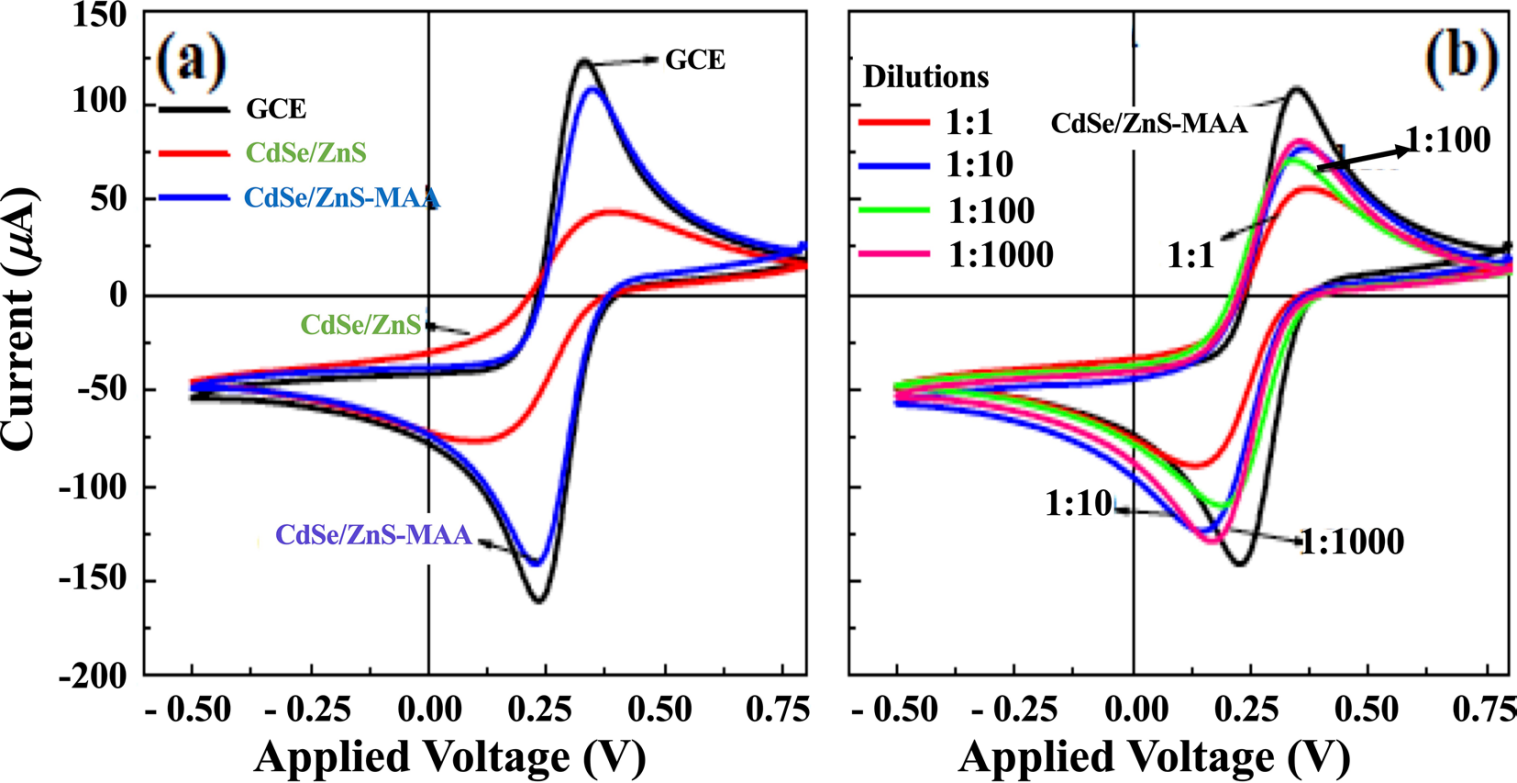
(**a**) CV scans of bare electrode (black), QDs (red) and MAA-QDs (blue) modified GC electrodes. (**b**) Variation in the CV of MAA-QDs (black) after conjugation with different dilutions of COx: 1:1 (red), 1:10 (blue), 1:100 (green), and 1:1000 dilution (pink) COx conjugated QDs.

The charge transfer mechanism during the conjugation was studied by cyclic voltammetry (CV) by using the glassy carbon (GC) electrode modified with COx-MAA-QDs. Figure 3a shows the CV scans of MAA-QDs modified GCE electrode taken at each step of modification (represented by different colors) at a scan rate 30 mVs^−1^ for bare (black color), CdSe/ZnS (red color) and MAA-CdSe/ZnS (blue color). The Fig. 3b represents the CV scans of COx conjugated MAA capped CdSe/ZnS QDs for four dilutions 1:1 (red color), 1:10 (blue color), 1:100 (green color), and 1:1000 (pink color). Also shown is the CV scan of the MAA-QDs (black color) as a reference. Well defined redox peaks of oxidation/reduction currents (Ipa and Ipc) at the respective potentials (Epa and Epc) were observed, which varied in magnitude with dilutions. It is well known that the redox peak currents are proportional to the flux of ions moving towards the electrode surface. During the chemical reaction transfer of charge takes place when positive bias is applied (i.e., forward scan) to the glassy carbon electrode, it oxidizes $\text{Fe}{(CN)}_{6}^{-4}$ and generates anodic current (Ipa) at the electrode. While reduction/cathodic current is generated during reverse or negative scan. The oxidation and reduction reactions are given in the following:

1 $$\text{Oxidation}{:}\;\;\text{ Fe}{(CN)}_{6}^{-4}\to \text{Fe}{(CN)}_{6}^{-3}+e$$

2 $$\text{Reduction}{:}\;\;\text{ Fe}{(CN)}_{6}^{-3}+e\to \text{Fe}{(CN)}_{6}^{-4}$$

In the forward scan $\text{Fe}{(CN)}_{6}^{-4}$ electrochemically oxidizes to generate $\text{Fe}{(CN)}_{6}^{-3}$ and in the reverse scan $\text{Fe}{(CN)}_{6}^{-3}$ is reduced back to $\text{Fe}{(CN)}_{6}^{-4}.$

The chemical reactions taking place at the electrode surface in various dilutions were responsible for variation in redox potentials and corresponding peak currents as observed in Fig. 3a,b during various stages of electrode modifications. The GC electrode modified with QDs showed a sharp decrease in the redox peak current, which was due to covering of QDs with TOPO that inhibited the charge transfer at the interface. The presence of long aliphatic chains of TOPO (an inorganic solvent) made it difficult for redox species to transfer charge toward the surface of CdSe/ZnS QDs. However, for MAA-CdSe/ZnS QDs modified GCE in alkaline pH led to the deprotonation of MAA that provided the path for transfer of charge and enhanced the redox peak currents (blue curve) shown in Fig. 3a. The conjugation of different dilutions of COx with the MAA-CdSe/ZnS QDs resulted in the increase in the redox peak currents as compared to TOPO-CdSe/ZnS QDs modified GCE (Fig. 3b). This implied the efficient charge transfer process through COx-MAA-QDs in various dilutions (effect of pH) between electrode surface and electrolytic solution that affected the PL intensity in various dilutions.

**Figure 4:**
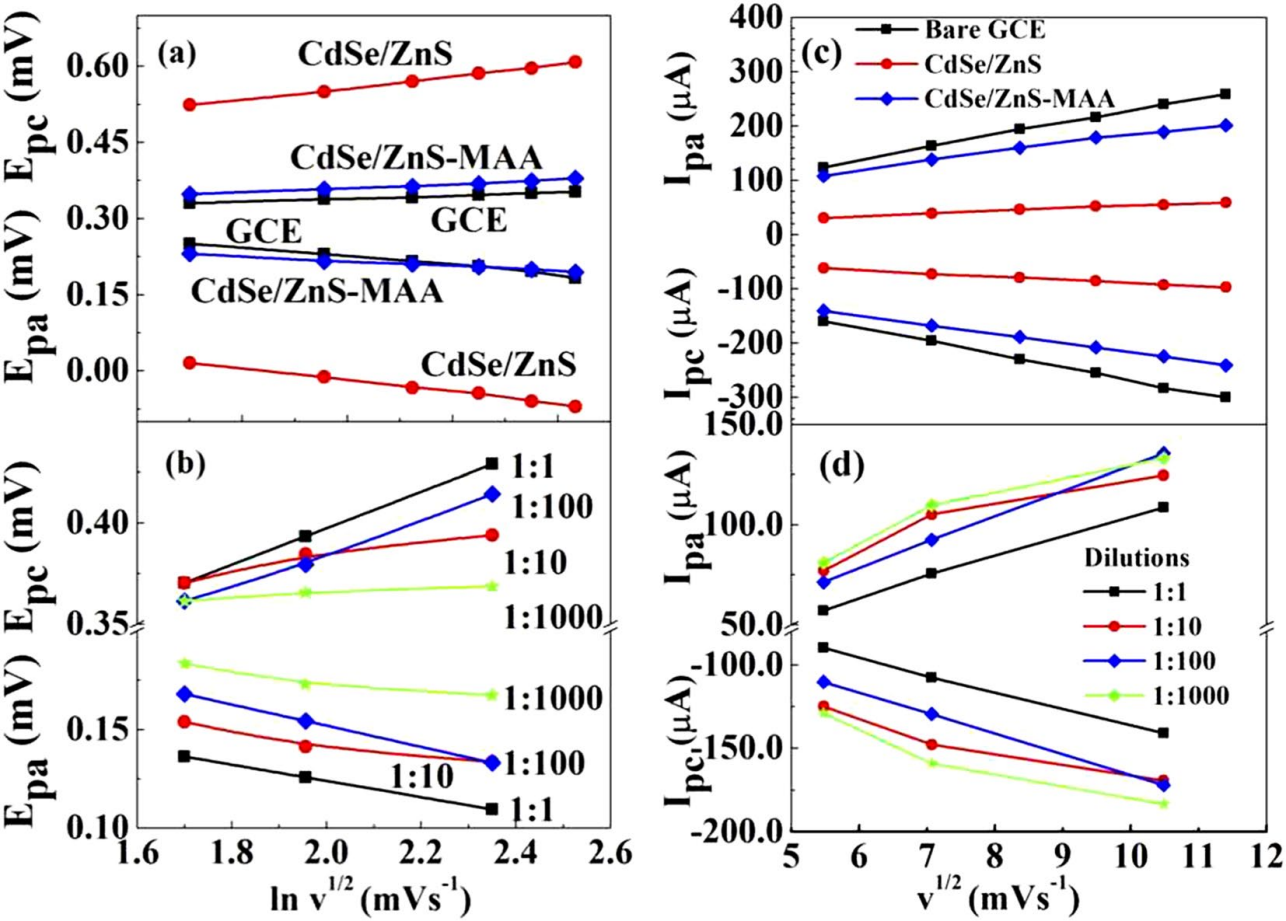
(**a**) E_pa_ and E_pc_ as a function of $ln\sqrt{v}$/mVs^−1^ for GCE (black cline), QDs (red line) and MAA-QDs (blue line) modified GCE.**(b)** E_pa_ and E_pc_ as a function of $ln\sqrt{v}$/mVs^−1^ for (**a**) 1:1, (**b**) 1:10, (**c**) 1:100, and (**d**) 1:1000 COx conjugated MAA-CdSe/ZnS QDs modified GCE. (**c**) *I*_pa_ and *I*_pc_ as a function of $\sqrt{v}$/mVs^−1^ for GCE (black line), QDs (red line) and MAA-QDs (blue line) modified GCE. (**d**) *I*_pa_ and *I*_pc_ as a function of $\sqrt{v}$/mVs^−1^ for 1:1 (black line), 1:10 (red line), 1:100 (blue line), and 1:1000 (green line) dilutions COx-MAA-QDs modified GCE.

The transfer kinetics between the electrode and redox species were further explored by CV measurements performed at various scan rates from 30 to 130 mVs^−1^. Figure 4a,b shows plots the E_pa_ and E_pc_ as a function of square root of scan rate at each step of electrode modification. The plots clearly demonstrated an increase in E_pa_ with a slope of 0.017 and decrease in E_pc_ with a slope of 0.037. There was a shift in potential and current with increasing scan rate as shown in Fig. 4a–d, so the reaction was quasi-reversible or irreversible^24^. The number of electrons transferred during each step was determined using Laviron equation^25,26^:

3 $$\frac{RT}{\text{slope}(\text{Ep}/{\text{ln}(v}^\frac{1}{2}) \text{) F}}= \upalpha n$$

where, ideal value of energy transfer coefficient $(\alpha =0.5$) was used for the determination of ‘*n*’ during redox reaction at each step of electrode modification.

The number of electrons calculated for GCE and CdSe/ZnS was 1 and MAA-CdSe/ZnS QDs was 2. This number increased in dilutions as for dilutions the electrons transferred 1:1 and 1:10 were 4 and for 1:100 and 1000 were 3. The electron transfer rate (slow/fast) during the anodic/cathodic reactions was determined through the electron diffusion coefficient (*D*_o_). The plot of ${I}_{pa}$ and *I*_pc_ as a function of $\sqrt{v}$ showed a linear dependence for all modified electrodes and all dilutions as shown in Fig. 4c,d, respectively, which reflected that the reaction was diffusion controlled. The linear behavior of *I*_p_ also satisfied the Randles–Sevcik relation given in Eq. (4)^26,27^:

4 $${I}_{p}=0.4461\; nFA{C}_{o}{\left(\frac{\alpha nFv{D}_{o}}{RT}\right)}^{-1/2}$$

where ${D}_{o}$ (cm^2^s^−1^) is the diffusion coefficient, ${I}_{p}$ $(\upmu\text{ A}$) represents the anodic/cathodic peak current in, *n* the number of electrons involved in redox reaction, F (C/mol) the faraday constant, A (cm^2^) the surface area of GCE, C_o_ (mol/cm^3^) is the concentration of electrolyte, *v* is the scan-rate, R is the universal gas constant and T the absolute temperature.

**Figure 5:**
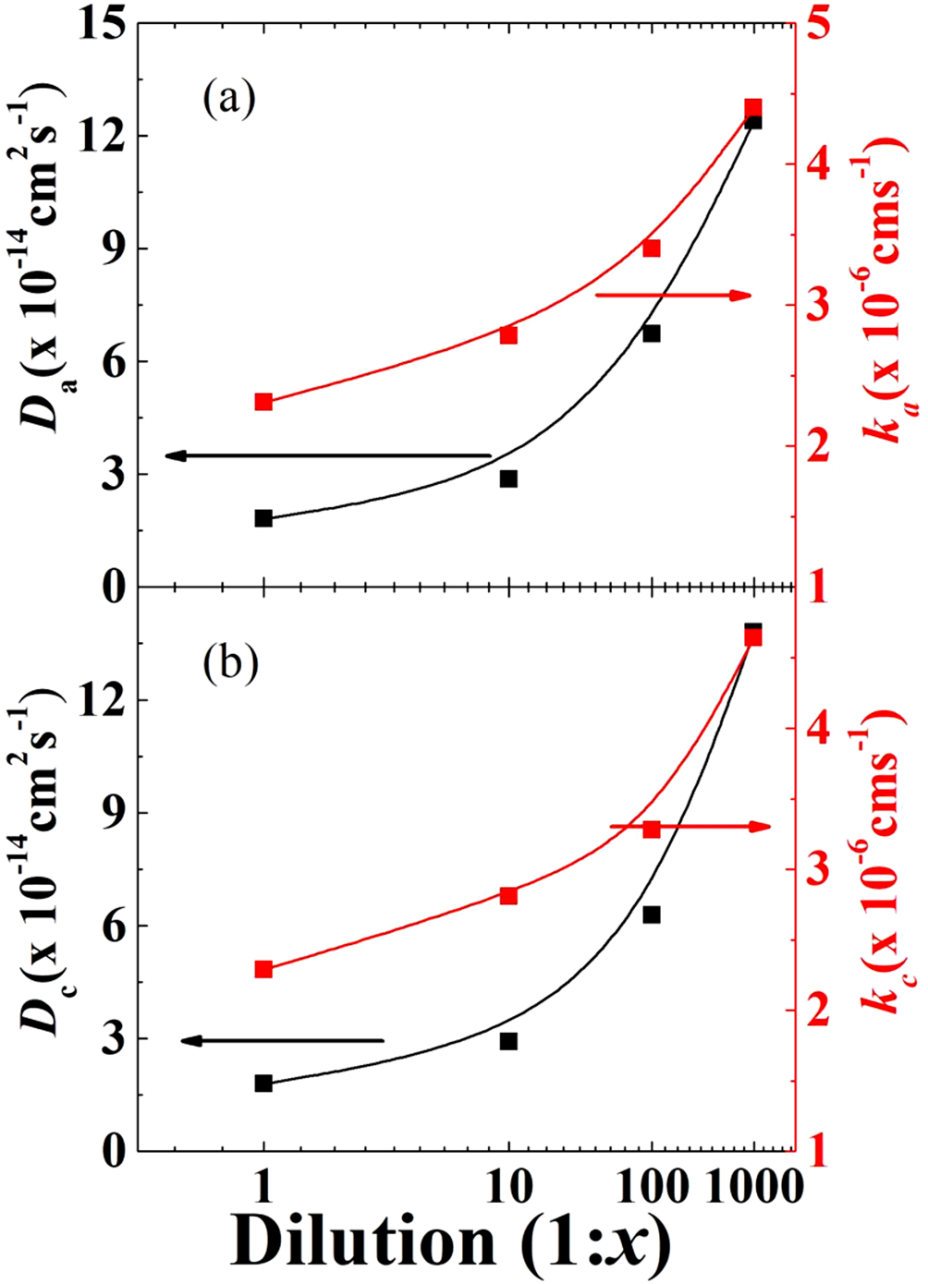
(**a**,**b**) Variation in diffusion coefficient *D*_*a*_*, D*_*c*_ and charge transfer rates *k*_*a*_*, k*_*c*_ for various dilutions of COx-MAA-QDs modified GC electrode as determined from CV measurements.

The diffusion coefficients (D_a_ during anodic current, D_c_ during cathodic current) as determined from Eq. (4) for the bare GC, QDs, MAA-QDs and Cox-MAA-QDs (1:1, 1:10, 1:100 and 1:1000 dilutions) modified electrodes are plotted in Fig. 5a,b (left vertical). It can be seen that the diffusion coefficient increased linearly with dilution. The diffusion coefficient for bare GCE was ~ 2 × 10^–14^ cm^2^/s and increased to 14 × 10^–14^ cm^2^/s in 1:1000 dilution. The increase in diffusion coefficient with increase in dilution was an indication of enhanced movement of redox species towards the electrode. Figure 5a,b (right vertical) also shows the transfer rate during anodic and cathodic currents, which increased with the increase in dilution. The charge transfer rate or rate constant (*k*_a_ during anodic current, *k*_c_ during cathodic current) depended on the thickness and surface coverage of the diffusion layer formed between the electrode and redox species. The thickness and surface area of diffusion layer formed at the interface between electrode and electrolyte species was found to depend on the scan rate. At slower scan rates the diffusion layer was thick, and consequently very small flux of ions moved from electrolytic solution to the electrode surface. On the contrary, faster scan rate made the electrode surface partially covered by the thin diffusion layer, which allowed more ion flux from electrolyte to the electrode surface. The increase in D_a_ and D_c_ and *k*_a_ and *k*_c_ showed that the diffusion layer became thin and partially covered the electrode surface with the increase in dilution.

**Figure 6:**
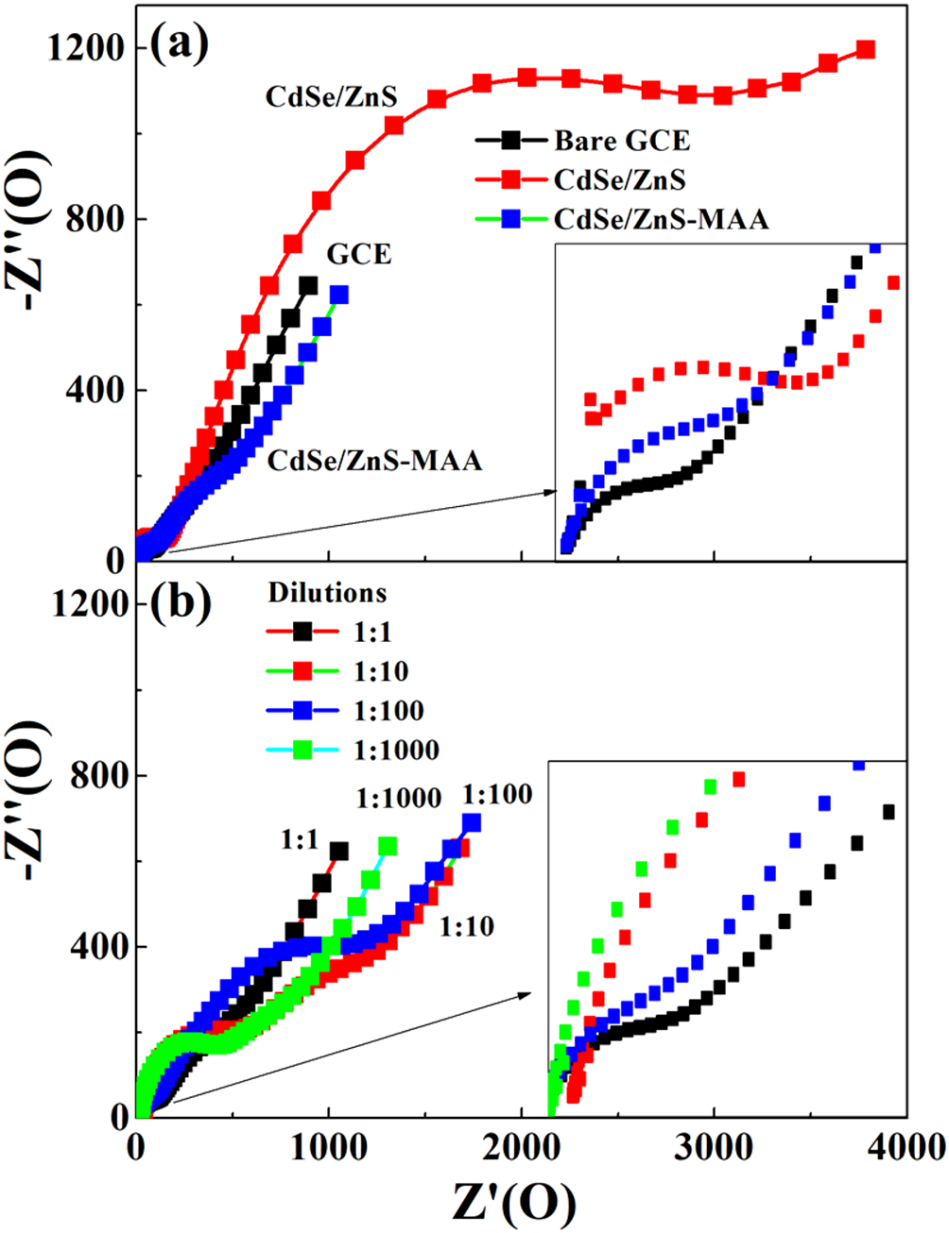
(**a**) Nyquist plot of bare (black color), QDs (red color) and MAA-QDs (blue color) modified GCE. (**b**) Nyquist plot of MAA-QDs (black color) after conjugation with different dilutions of COx: 1:1 (red color), 1:10 (blue color), 1:100 (green color), and 1:1000 dilution (pink color) COx conjugated QDs. The insets show the high frequency part of the plot.

The electrochemical impedance spectroscopy (EIS) was carried out to determine diffusion coefficients and charge transfer rates in various dilutions. This was used to find the charge transfer resistance, double layer capacitance and diffusion resistance of the system (solution and modified electrode). Figure 6a,b show the EIS spectra of electrodes coated with bare QDs, MAA-QDs and Ab-MAA-QDs in various dilutions. The Nyquist plots for the bare GCE consisted of a small semi-circle at high frequencies followed by a straight line at low frequencies. Generally, the semi-circle represented the resistance offered by the electrolytic species to the electrode due to formation of a capacitive layer and straight line represented the diffusion of redox species directly to electrode. The MAA-QDs and Ab-MAA-QDs modified electrodes showed the presence of two semi-circles as shown in Fig. 6a,b, one a broad one at low frequencies and other a narrow one at high frequencies. The high frequency semi-circle was due to the reaction occurring between the electrode and electrolytic solution. While the low frequency semi-circle was ascribed to the absorption of charges on electrode surface creating a second channel for current^28,29^.

**Figure 7:**
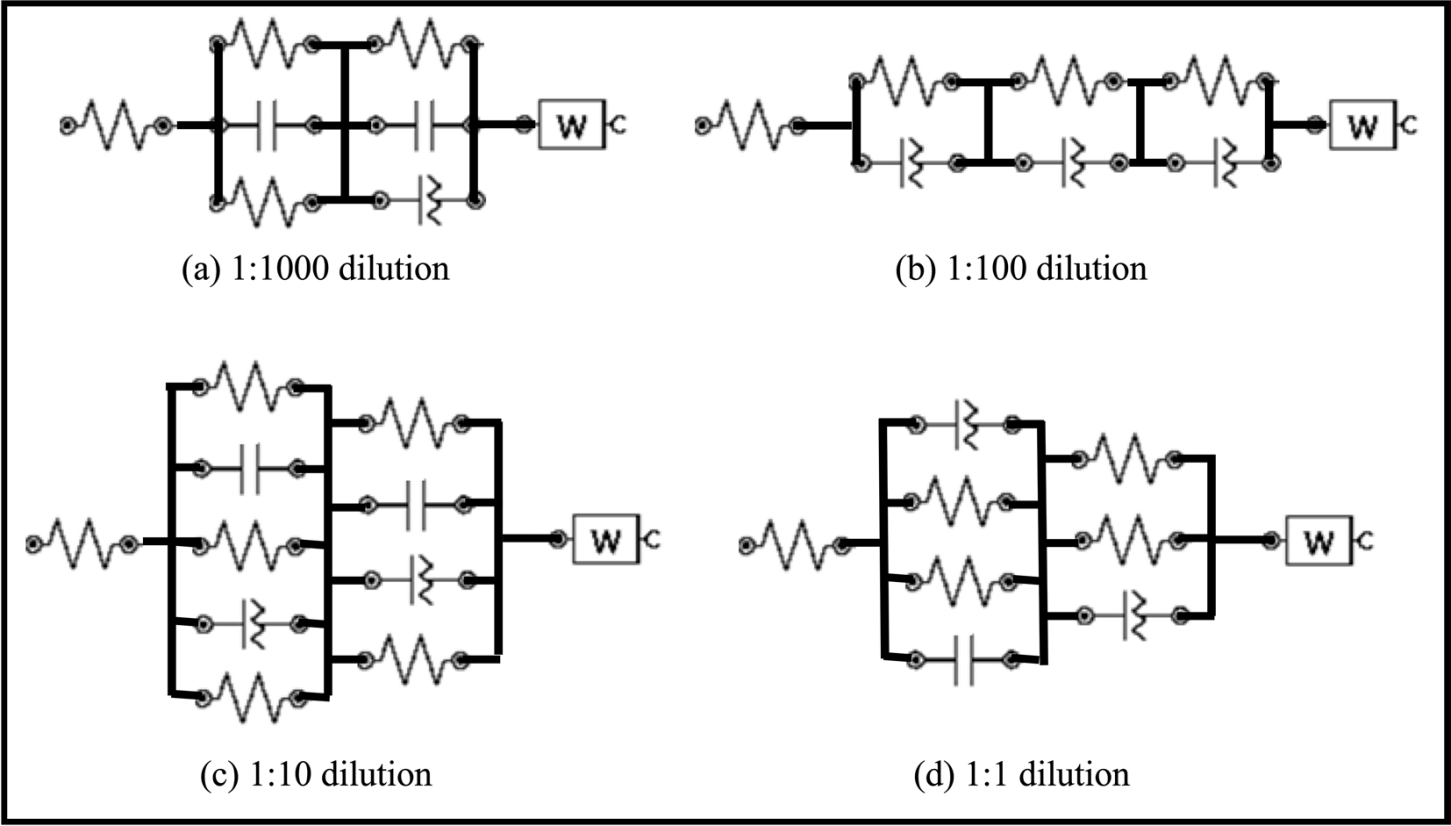
Equivalent circuit for CdSe/ZnS-MAA-EDC/NHS-COX modified GCE in (**a**) 1:1000, (**b**) 1:100, (**c**) 1:10 and (**d**) 1:1 dilutions.

The equivalent circuits of the modified electrodes are drawn in Fig. 7a–d for various dilutions (a) 1:1; (b) 1:10; (c) 1:100; and (d) 1:1000, which consisted of electrolytic solution resistance (R_s_), charge transfer resistance (R_ct_) of redox, double layer capacitance (C_dl_) and resistance (R_dl_), constant phase element (CPE) and Warburg element (W). The extracted values of R_s_, R_ct_ are shown in Table 1. An increase in Rs and Rct was observed in CdSe/ZnS QDs modified GCE compared to bare GCE, which dropped drastically in the MAA-QDs modified electrodes (Table 1). This was attributed to the MAA coating, which assisted exchange of species (electrons) between the electrode and the electrolytic solution due to the COO^−^ group of MAA. The systematic variation in the values of Rs and Rct suggested that the presence of ions on the surface of electrode played a key role in the conduction of ions. The values were small in the stock solution, which increased with the increase of dilution and started decreasing with the increase in dilutions. This was attributed to the transition from the state of protonated surface to the state of deprotonated surface. In either case, the values of Rs and Rct remained small.

**Table 1:** Equivalent circuit values by fitted Nyquist plot for 1:1000, 1:100, 1:10 and 1:1 diluted Cox conjugated CdSe/ZnS QDs.

Electrode modification	R_s_ ($\Omega$)	R_ct_ ($\Omega$)
Bare GCE	29.9–45.4	69.0–662
CdSe/ZnS	100–418.3	1305–6656
CdSe/ZnS-MAA	5.90–11.0	147–193
CdSe/ZnS-MAA-COx (1:1 dilution)	8.65	1890
CdSe/ZnS-MAA-COx (1:10 dilution)	24.5	1150
CdSe/ZnS-MAA-COx (1:100 dilution)	18.5	557
CdSe/ZnS-MAA-COx (1:1000 dilution)	2.26	228

The charge transfer resistance Rct showed an abrupt increase from bare GCE to QD coated electrode. However, it dropped significantly for MAA-QDs modified GC electrode and then increased for 1:1 diluted Cox-MAA-QDs modified GC electrode. Eventually dilutions showed its effect as Rct dropped again significantly from 1890 Ω for 1:1 dilution to 228 Ω for 1:1000 dilution. The significant drop in Rct was due to the occurrence of the Faradic reaction in large dilutions as it provided more alkaline environment and reduced the solution resistance. The presence of CPE (in micro range) showed the presence of adsorbed molecules and surface roughness during all steps of electrode modification. The conductance due to CPE and Warburg elements were comparable in all steps of modification, which confirmed that the electrochemical reaction was diffusion controlled only.

## Conclusions

The CdSe/ZnS core–shell quantum dots were synthesized via organometallic method for use in the optical detection of COx antibodies. The core–shell QDs were made water-soluble by replacing the TOPO with MAA. The COOH group present on the surface of MAA coated QDs was activated by EDC/NHS coupling. The activated MAA–CdSe/ZnS QDs were then conjugated with serially diluted COx antibody. MAA-QDs showed poor PL intensities, which were attributed to escape of photo-excited carriers from QDs to OMO/LUMO states of MAA. However, enhanced PL response was observed from COx–MAA–QDs conjugated at high pH (high dilutions 1:100 and 1:1000). This was due to passivation of HOMO and LUMO states of MAA in the presence of COx. The electrochemical analysis revealed enhanced diffusion of redox species towards the electrode surface for higher dilutions of COx. Furthermore, the gradual enhancement in rate constant with the increase in dilution depicted that 1:1000 COx dilution allowed the faster electron transfer in contrast to other dilutions. The small values of R_s_ and R_ct_ for 1:1000 dilution also confirmed that Faradic reaction occurs faster. The present study confirmed the role of dilutions in critical in charge transfer processes and detection of antibodies such as sensitive detection of cholesterol.

## References

[1] Park NM, Kim TS, Park SJ (2001). Band gap engineering of amorphous silicon quantum dots for light-emitting diodes. Appl. Phys. Lett..

[2] Bruchez M, Moronne M, Gin P, Weiss S, Alivisatos AP (1998). Semiconductor nanocrystals as fluorescent biological labels. Science.

[3] Stojanovic MN, Stefanovic D (2003). A deoxyribozyme-based molecular automaton. Nat. Biotechnol..

[4] Huang CP, Li YK, Chen TM (2007). A highly sensitive system for urea detection by using CdSe/ZnS core–shell quantum dots. Biosens. Bioelectron..

[5] Goldman ER, Medintz IL, Hayhurst A, Anderson GP, Mauro JM, Iverson BL, Mattoussi H (2005). Self-assembled luminescent CdSe–ZnS quantum dot bioconjugates prepared using engineered poly histidine terminated proteins. Anal. Chim. Acta..

[6] Smith AM, Nie S (2010). Semiconductor nanocrystals: structure, properties and band gap engineering. Ace. Chem. Res..

[7] Carrion CC, Cardenas S, Simonet BM, Valcarcel M (2009). Quantum dots luminescence enhancement due to illumination with UV/Vis light. Chem. Commun..

[8] Reiss P, Bleuse J, Pron A (2002). Highly luminescent CdSe/ZnSe core/shell nanocrystals of low size dispersion. Nano Lett..

[9] Fu A, Gu W, Boussert B, Koski K, Gerion D, Manna L, Gros ML, Larabell C, Alivisatos AP (2007). Semiconductor quantum rods as single molecule fluorescent biological labels. Nano Lett..

[10] Chaudhry M, Lim DK, Qamar R, Bhatti AS (2018). The adverse role of excess negative ions in reducing the photoluminescence from water soluble MAA–CdSe/ZnS quantum dots in various phosphate buffers. Phys. Chem. Chem. Phys..

[11] Chaudhry M, Rehman MA, Gul A, Qamar R, Bhatti AS (2017). The effect of varied pH on the luminescence characteristics of antibody–mercaptoacetic acid conjugated ZnS nanowires. Chem. Phys..

[12] Kim KE, Kim T, Sung YM (2012). Fluorescent cholesterol sensing using enzyme-modified CdSe/ZnS quantum dots. J. Nanoparticle Res..

[13] MacLachlan J, Wotherspoon ATL, Ansell RO, Brooks CJW (2000). Cholesterol oxidase: Sources, physical properties and analytical applications. J. Steroid Biochem. Mol. Biol..

[14] Staff M. C., Cholesterol test: sorting out the lipids, MayoClinic.com (2007). https://www.mayoclinic.org/tests-procedures/cholesterol-test/about/pac-20384601.

[15] Tatsuma T, Watanabe T (1991). Oxidase/peroxidase bilayer-modified electrodes as sensors for lactate, pyruvate, cholesterol and uric acid. Anal. Chim. Acta..

[16] Lin X, Ni Y, Kokot S (2016). Electrochemical cholesterol sensor based on cholesterol oxidase and MoS2-AuNPs modified glassy carbon electrode. Sens. Actuators B Chem..

[17] Cortez J, Vorobieva E, Gralheira D, Osório I, Soares L, Vale N, Pereira E, Gomes P, Franco R (2011). Bionanoconjugates of tyrosinase and peptide-derivatised gold nanoparticles for biosensing of phenolic compounds. J. Nanoparticle Res..

[18] Dabbousi BO, Viejo JR, Mikulec FV, Heine JR, Mattoussi H, Ober R, Jensen KF, Bawendi MG (1997). (CdSe)ZnS core-shell quantum dots: Synthesis and characterization of a size series of highly luminescent nanocrystallites. J. Phys. Chem. B..

[19] Georgin M, Carlini L, Cooper D, Bradforth DE, Nadeau JL (2013). Differential effects of b-mercaptoethanol on CdSe/ZnS and InP/ZnS quantum dots. Phys. Chem. Chem. Phys..

[20] Aldana J, Lavelle N, Wang Y, Peng X (2005). Size-dependent dissociation pH of thiolate ligands from cadmium chalcogenide nanocrystals. J. Am. Chem. Soc..

[21] Poderys V, Matulionyte M, Selskis A, Rotomskis R (2011). Interaction of water-soluble CdTe quantum dots with bovine serum albumin. Nanoscale Res. Lett..

[22] Tournon J, Kuntz E, El-bayoumi MA (1972). Fluorescence quenching in phenylalanine and model compounds. Photochem. Photobiol..

[23] Vo NT, Ngo HD, Vu DL, Duong AP, Lam QV (2015). Conjugation of *E. coli* O157:H7 antibody to CdSe/ZnS quantum dots. J. Nanomater..

[24] Aristov N, Habekost A (2015). Cyclic voltammetry—A versatile electrochemical method investigating electron transfer process. World J. Chem. Educ..

[25] Laviron E (1979). General expression of the linear potential sweep voltammogram in the case of diffusionless electrochemical systems. J. Electroanal. Chem. Interfacial Electrochem..

[26] Bard A (2001). Electrochemical Methods: Fundamentals and Applications.

[27] Massot L, Chamelot P, Bouyer F, Taxil P (2002). Electrodeposition of carbon films from molten alkaline fluoride media. Electrochim. Acta.

[28] Yezer BA, Khair AS, Sides PJ, Prieve DC (2016). Determination of charge carrier concentration in doped nonpolar liquids by impedance spectroscopy in the presence of charge adsorption. J. Colloid Interface Sci..

[29] Wang L, Zhao J, He X, Gao J, Li J, Wan C, Jiang C (2012). Electrochemical impedance spectroscopy (EIS) study of LiNi_1/3_Co_1/3_Mn_1/3_O_2_ for Li-ion Batteries. Int. J. Electrochem. Sci..

